# Genetics and Plasticity Are Responsible for Ecogeographical Patterns in a Recent Invasion

**DOI:** 10.3389/fgene.2022.824424

**Published:** 2022-03-11

**Authors:** Katarina C. Stuart, William B. Sherwin, Adam P.A. Cardilini, Lee A. Rollins

**Affiliations:** ^1^ Evolution and Ecology Research Centre, School of Biological, Earth and Environmental Sciences, UNSW Sydney, Sydney, NSW, Australia; ^2^ School of Life and Environmental Sciences, Deakin University, Waurn Ponds, VIC, Australia

**Keywords:** Sturnus vulgaris, European starling, invasive species, rapid adaptation, phenotype

## Abstract

Patterns of covariation between phenotype and environment are presumed to be reflective of local adaptation, and therefore translate to a meaningful influence on an individual’s overall fitness within that specific environment. However, these environmentally driven patterns may be the result of numerous and interacting processes, such as genetic variation, epigenetic variation, or plastic non-heritable variation. Understanding the relative importance of different environmental variables on underlying genetic patterns and resulting phenotypes is fundamental to understanding adaptation. Invasive systems are excellent models for such investigations, given their propensity for rapid evolution. This study uses reduced representation sequencing data paired with phenotypic data to examine whether important phenotypic traits in invasive starlings (*Sturnus vulgaris*) within Australia appear to be highly heritable (presumably genetic) or appear to vary with environmental gradients despite underlying genetics (presumably non-heritable plasticity). We also sought to determine which environmental variables, if any, play the strongest role shaping genetic and phenotypic patterns. We determined that environmental variables—particularly elevation—play an important role in shaping allelic trends in Australian starlings and may also reinforce neutral genetic patterns resulting from historic introduction regime. We examined a range of phenotypic traits that appear to be heritable (body mass and spleen mass) or negligibly heritable (e.g. beak surface area and wing length) across the starlings’ Australian range. Using SNP variants associated with each of these phenotypes, we identify key environmental variables that correlate with genetic patterns, specifically that temperature and precipitation putatively play important roles shaping phenotype in this species. Finally, we determine that overall phenotypic variation is correlated with underlying genetic variation, and that these interact positively with the level of vegetation variation within a region, suggesting that ground cover plays an important role in shaping selection and plasticity of phenotypic traits within the starlings of Australia.

## Introduction

Phenotypic trends within a species are often driven by the environment. Patterns of covariation between phenotype and environment are presumed to be reflective of local adaptation or plasticity, and translate to a meaningful influence on an individual’s overall fitness within that specific environment (Wainwright and Reilly 1994). Across many different ecological systems, a plethora of ecogeographical rules describe biologically significant trends between phenotypic traits and specific environmental measures, or even go as far as to extend to interspecific relationships and assemblage patterns (Gaston et al., 2008). Tolerance to temperature and other climate variance shifts are inherently important for a population to withstand extinction (Sekercioglu et al., 2008). Understanding how ecogeographical trends may arise or shift, and on what biological timescale, are imperative to understanding long term evolution of species (Robinson and Quinn 1988). Such questions are of increasing concern as climate change research seeks to understand how climate change may cause populations to undergo evolutionary changes (Gardner et al., 2011; Oostra et al., 2018; Duffy and Jacquemyn 2019).

While the relationship between phenotype and environment may be hard coded in the genome of an organism, it may also be a result of plasticity. Plasticity, the ability of an organism to produce different phenotypes in response to environmental variation but with the same genetic basis, is fundamental to explain phenotypic diversity within a species (West-Eberhard 1989). Overall phenotype is a result of underlying gene-environment interactions, in which plasticity plays an important role (Li et al., 2018). Therefore, plasticity encapsulates both heritable adaptive conditional responses (gene-environment interactions), as well as non-heritable environmentally produced variation (West-Eberhard 1989). There is much discussion on the role of plasticity in evolution (Sommer 2020; Uller et al., 2020), with considerations such as accommodation (West-Eberhard 2005) and canalisation (Debat and David 2001) as proposed mechanisms for selection on plastic traits to be translated into evolutionary change. An alternative viewpoint on plasticity is that plasticity itself may shield the genome from selective forces, because selection for or against a phenotype may not directly translate to selection for or against specific genetic variants (DeWitt et al., 1998). This is further complicated by the fact that plasticity itself is a trait that may be under selection (Nussey et al., 2005). Doubtless, the role of plasticity in a species evolution is nuanced and will be taxon or even population specific.

Invasive species are excellent model systems in which to investigate questions regarding heritable and non-heritable environmental effects on phenotype. Invasive species’ persistence relies in part on rapid adaptation, and plasticity acts as an immediate means through which an invasive species may be able to survive in their novel environment (Richards et al., 2006; Fox et al., 2019). Understanding the relationship between environmental variables shaping genetic patterns and the resulting phenotypes may be essential to better understand why a particular species has become such a successful invader (Caño et al., 2008). Native range populations may be close to equilibrium, and this may confound such investigations in these populations. In contrast, invasive populations are likely far from equilibrium and so enable the study of how a novel environment may cause rapid shifts in genetics and phenotype. Such research provides information not just for invasion management, but also may be used to investigate phenotypic shifts during alternate environmental projections expected under climate change. Patterns of climate induced ecogeographical patterns have been widely studied in birds (Buskirk et al., 2010; Goodman et al., 2012; Delhey et al., 2019), and determining heritable and non-heritable influences on these patterns will be invaluable for future scenario modelling and preserving native populations.

The European starling (*Sturnus vulgaris*, hereafter the starling) is a near-globally invasive passerine, and was introduced to Australia in the mid-late 19th century from their native range in Eurasia (Jenkins 1977). Previous research indicates that the invasive population of starlings in Australia has distinct population genetic structure (Rollins et al., 2009, 2011; Stuart et al., 2021a). The several major and geographically separate introduction sites along the eastern and southern Australian coastlines likely resulted in genetic footprints of founding individuals forming one southern and one eastern major genetic cluster, despite ongoing geneflow between the regions (Rollins et al., 2009; Stuart et al., 2021b). Previously, environmental distance between sampling sites was not found to significantly correlate with genetic distance (isolation by environment, IBE), while the geographic distance between sample sites was found to be a predictor of genetic distance (isolation by distance, IBD) (Stuart et al., 2021b).

However, these patterns in IBE and IBD do not exclude a role for environment in shaping the population genetics of Australian starlings—indeed it would be reasonable to expect (due to large differences from native range climate) at least some aspects of the novel invaded environment would have impacted the genetics of this species. Cardilini et al. (2016) found that body size (mass) and beak surface area varied with environmental temperature gradients. This may indicate that environmentally shaped adaptive patterns may be more nuanced (e.g., occurring in only a small number of genes, or being non-linear) or, alternatively, that much of the environmentally correlated phenotypic patterns are due primarily to plasticity.

It is important here to note that by studying morphological variation within the invaded Australian range, we may observe how original genetic diversity from the native range (from founding individuals) was acted upon to elicit adaptive phenotypic change, or alternatively which phenotypic traits were altered irrespective of the genetics, shedding light on plastic developmental responses in this species. With concern over plummeting starling numbers within the native range (Smith et al., 2012; Heldbjerg et al., 2016), we may use such findings to better understand how and to what extent starlings can adapt to shifting native range climates in the face of climate change. However, analysis of genetic and environmental effects of phenotypes within an invasive range do pose technical challenges, owing to the demographic nature of such populations. Ideally, assessment may reveal that phenotype varies only with genetic gradients (and particular genotypes, thus appearing to be heritable), or alternatively vary only with environmental gradients (thus appearing to be plastic). Phenotype might even depend upon interaction of genetics and environment, suggesting a combined role for heritability and plasticity. However, it is possible that genetics and environment might covary, either due to evolutionary processes such as selection, or neutral process such as demographic effects during post-introduction range expansion. In such cases the relationship between genetics, environment, and phenotype cannot be discerned using correlation studies alone, but they do facilitate the first step towards better understanding the mechanisms behind rapid phenotypic change within invasive ranges.

The present study uses reduced representation sequencing data with paired phenotypic, ecological, and spatial data to examine important phenotypic traits in Australian starlings. Specifically, we seek to answer four key aims. Firstly, we ask what environmental variables appear to be shaping the overall allelic frequency landscape. We expect that temperature and precipitation play a vital role due to major differences in these variables between Australia and the native range. Next, we examine the heritability of different phenotypic traits and how this is partitioned across the chromosomes. We predict that few traits will be heritable, and that many of these heritable traits will be polygenic and so will show a linear relationship between explained variance per chromosome and chromosome size. We then ask which SNPs are correlated with phenotype, and then examine the amount of genetic variance in these SNPs that is explained by key environmental predictors. Finally, we assess genetic, phenotype, and environmental relationships more broadly by examining patterns in variance. We investigate whether phenotypic variance correlates with genetic variance and/or environmental variance, and if there are any range-edge effects. We predict that phenotypic variance would be lower at the range edge due to genetic drift in these smaller populations, and that phenotype and genetic variance would be positively correlated.

## Materials and Methods

### Genetic Sequencing Data, Variant Calling, and Filtering

We used published genetic data produced using reduced representation sequencing (Stuart et al., 2021b; BioProject ID 655259). Briefly, a total of 499 individuals underwent Genotype by Sequencing (GBS; Elshire et al., 2011). Samples were collected between 2003–2011 (approximately 4 generations: Higgins et al., 2006; Rollins et al., 2016) from the southern and eastern portions of the Starlings’ Australian range. We aligned the demultiplexed files to the starling reference genome *S. vulgaris* vAU1.0 (Stuart et al., 2021a; accession GCF_ JAGFZL000000000) using BWA *mem* v0.7.17 (Li 2013), then converted the SAM files into sorted BAM format using samtools v1.10 (Li et al., 2009). We added read groups using Picard v2.18.26 (Picard toolkit 2019), used Stacks *gstacks* to genotype individuals, and called variants using *populations* (Rochette et al., 2019).

**FIGURE 1:**
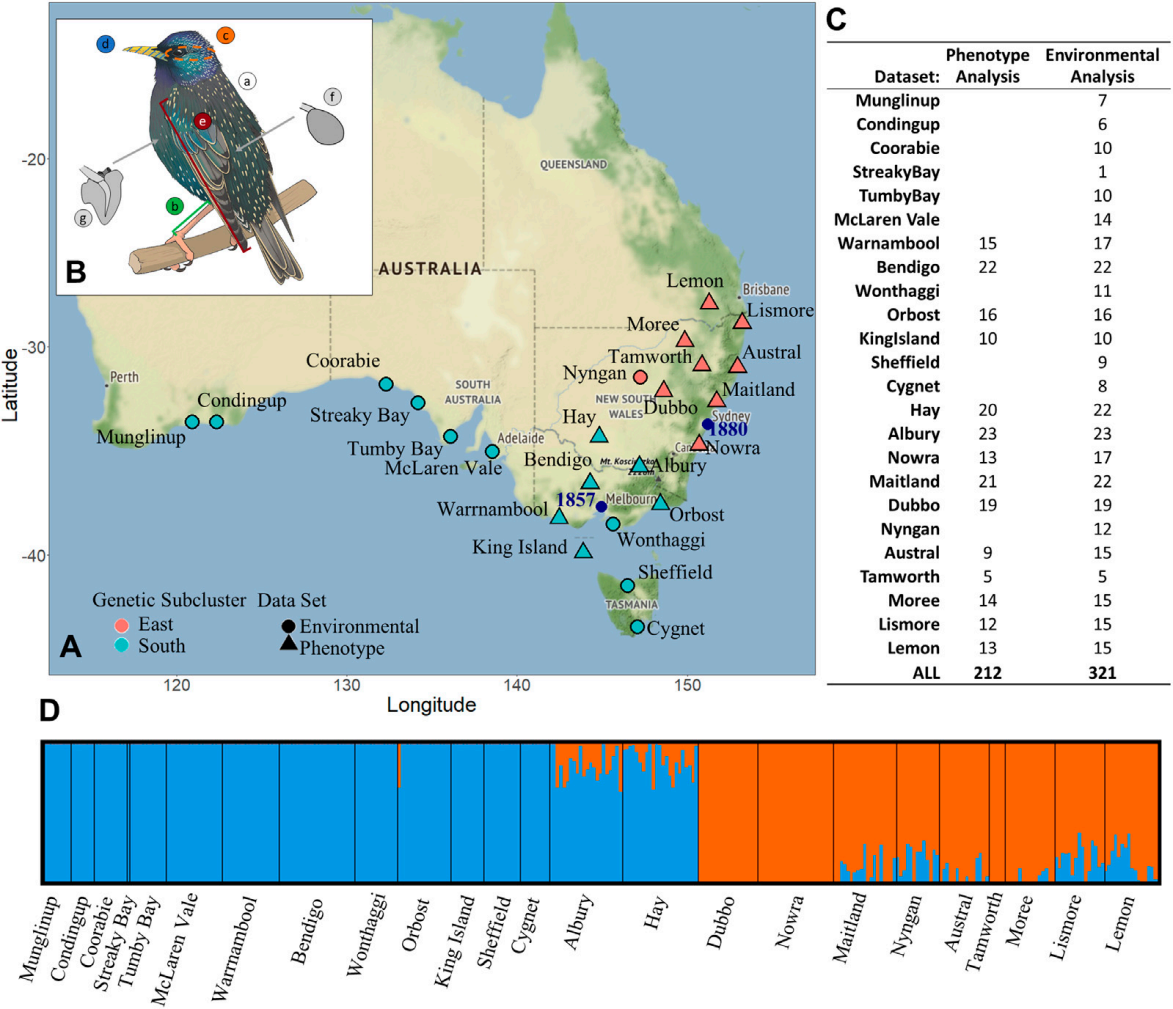
*Sturnus vulgaris* sample site and phenotypic sample site information including panel **(A)** starling genetic data sample sites, with colour denoting which genetic subcluster the sample site belongs to (orange = eastern, blue = southern), while shape shows which analysis that sample site was included in (triangle = phenotype and environmental, circle = environmental only). Panel **(B)** depicts the seven phenotypic traits that were measured (A: mass, B: tarsus length, C: head antero-posterior cross section, D: beak surface area, E: wing length, F: spleen mass, G: heart mass), and panel **(C)** shows the number of individuals used in either phenotype analysis (see *Heritability and Chromosomal Partitioning of Phenotypic Variance*, *Genome-Phenotype Association Test*, and *Variance in Phenotype, Genetics, and Environment* sections) or environmental analysis (*Environmental and Spatial Associations With Genome Wide Genetic Variation* section). Panel **(D)** depicts an admixture ancestry analysis for all 321 samples used in the environmental analysis study, used to determine group membership in panel a.

**FIGURE 2:**
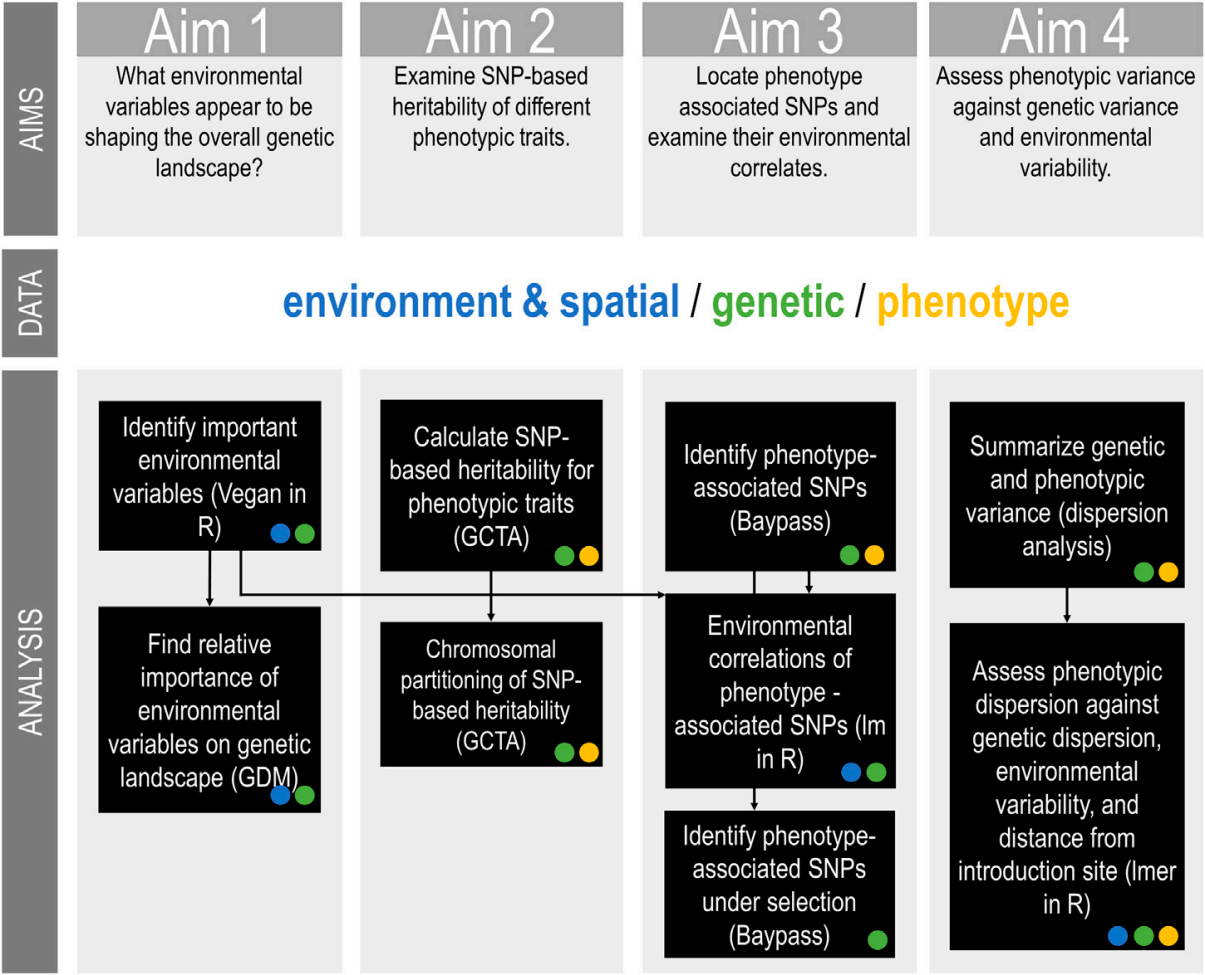
Australian *Sturnus vulgaris g*enetic, phenotypic, and environmental analysis methods summary, with the type of input data (environmental/spatial, genetic, or phenotype) for each analysis step indicated as a circle within the relevant box. Black arrows indicate when output of previous analysis is used as input in a subsequent analysis step.

We used VCFtools to quality filter genetic data for a maximum locus missingness of 50% (--max-missing 0.5), a minor allele frequency of 0.05 (--maf 0.05, because this study does not focus on “rare” alleles), a minimum allele depth of 2 (--min-meanDP 2), a maximum allele depth of 50 (--max-meanDP 50), an allele number of 2 (--min-alleles 2 --max-alleles 2), and a minimum genotype quality score of 15 (--minGQ 15). We then pruned the SNP data set for linkage using BCFtools *prune* for SNPs with linkage values of less than 0.6 in 1,000 bp sliding windows (-l 0.6 -w 1,000). We assessed the per-individual missingness levels, and any individual that was missing sequencing data in more than 50% of SNP sites was excluded from the final data set. This produced a data set with 111,037 SNPs from a total of 321 individuals across 24 sample sites (Figures 1A,C). This data set was used to explore genetic and environmental associations (*Variance in Phenotype, Genetics, and Environment (Aim 4)* section; Figure 2: aim 1). We used the program Admixture v1.3.0 (Deng et al., 2001) to conduct ancestry admixture analysis for K = 2, to be compared to previous admixture analysis from Stuart *et. al.* conducted on the same data set but without a reference genome (Stuart et al., 2021b).

We produced a second genetic data set, which was a subset of the genetic dataset above containing only those individuals for which we had phenotypic data (see below), for use in analysis that involved phenotype (Figure 2: aims 2–4). For this analysis, we refiltered the initial VCF file produced by stacks *populations*. All filtering steps remained the same as those detailed above, however prior to quality filtering we removed individuals from sample sites with less than five total individuals with corresponding phenotypic data (see *Phenotypic and Environmental Sample Data* section). This produced a data set with 128,217 SNPs from a total of 212 individuals across 14 sample sites (Figure 1C).

### Phenotypic and Environmental Sample Data

The phenotype data that we used in this study was source from Cardilini et al. (2016), along with additional, previously unpublished phenotypic trait measurements that were taken from the same birds at the same time. This phenotype data was collected in between May 2011 and October 2012. We only retained individuals in the data set that had corresponding genetic data post quality filtering and had data for at least four out of the seven phenotypic measurements shown in Figure 1B. Phenotypic measurements included: mass (g, N = 212), tarsus length (mm, N = 212), head antero-posterior cross section (mm^2^, N = 143, calculated using (head and beak length−beak length)×head width×π ), beak surface area (mm^2^, N = 199, calculated as per Greenberg et al. (2012) using the formula $\left(\frac{beak\;depth+beak\;width}{4}\times beak\;length\right)$ ), wing length (mm, N = 201), spleen mass (g, N = 178), and heart mass (g, N = 184). We allometrically corrected these phenotypic data for size-related collinearity, by regressing the phenotypic trait against the individual’s tarsus length, and the residuals were retained (to be used as the new size-corrected measures) if the correlation was significant (though note some caution in using residual based allometric corrections: Freckleton 2002). We chose tarsus length because it is an accurate predictor of body size in birds (Senar and Pascual 1997). We found a total of four phenotypic measures to be significantly correlated with tarsus length: mass, head antero-posterior cross section, beak surface area, and wing length (Supplementary Figure S1; Supplementary Table S1) and these were size corrected for the final data set (Supplementary Figure S2).

### Environmental and Spatial Associations With Genome Wide Genetic Variation (Aim 1)

To quantitatively assess the contributions of spatial and environmental variables to the overall genetic variation within the Australian range (Figure 2: aim 1), we used a gradient forest approach. Gradient forest (GF) is a nonlinear, nonparametric machine-learning regression tree approach that enables the partitioning of nonlinear associations of allelic variable data to spatial and environmental variables. We conducted all environmental and spatial association analysis in R v3.5.3. It has previously been demonstrated in this study system that overall population substructure strongly reflects historical demographic patterns and other spatial processes (common in invasive populations), alongside the patterns resulting from natural selection and environmentally induced substructure (Stuart et al., 2021b). The GF approach can account for the presence of spatial processes through the use of Moran’s eigenvector map variables (Dray et al., 2006), thereby enabling the calculation of the unique contribution of each environmental variable to the allelic patterns. To do this, we computed a distance matrix of the sample longitude and latitude values using the dist() function, and then used the function pcnm() in Vegan (Oksanen et al., 2019) to conduct Principal Coordinate of Neighbourhood Matrix (PCNM) analysis to transform the spatial distances to data suitable for regression. Half of the positive PCNM axis values were retained to include in the GF model (n = 10) (Manel et al., 2010). Environmental variables were selected by retrieving all BioClim variables (Bio01-Bio19) and altitude (Worldclim (Fick and Hijmans, 2017), obtained through raster package (Hijmans and van Etten, 2012). To create a dataset of environmental variables with minimised collinearity, we then applied the pairs.panel() function in vegan to iteratively remove variables that were highly colinear with many other variables. The final environmental variable list consisted of: bio1 (annual mean temperature), bio3 (isothermality), bio4 (temperature seasonality), bio8 (mean temperature of wettest quarter), bio9 (mean temperature of driest quarter), bio12 (annual precipitation), bio14 (precipitation of driest month), bio15 (precipitation seasonality), bio18 (precipitation of warmest quarter), bio19 (precipitation of coldest quarter), elevation, and mnNDVI (mean normalized difference vegetation index).

We conducted GF analysis using the gradientForest() function in gradientforest v 0.1–24 (Ellis et al., 2012). The spatial PCNM variables and the environmental variables were used as the predictor variables, with the SNP data functioning as the response variables. The function was run on default settings (ntree = 500, maxLevel = maxLevel, trace = T, corr. threshold = 0.50). We used the inbuilt gradientforest plot() function to assess the varying importance of predictor spatial PCNMs or environmental variables, and to visualise allelic composition changes along the important environmental gradients. We then used the predict() function, which predicts compositional patterns across geographic space. We used the GF model produced in the above analysis to predict genetic patterns across the gridded environmental variables across Australia, and visualised the first three principal components produced when this was parsed into the function prcomp().

### Heritability and Chromosomal Partitioning of Phenotypic Variance (Aim 2)

To calculate phenotypic trait SNP-based heritability values, and partition genetic variance across chromosomes (Figure 2: aim 2), we used GCTA (Yang et al., 2011a) following published pipelines (Yang et al., 2011b). GCTA generates per-individual genetic relationship matrixes (GRMs), which estimate the genetic distance between individuals using provided SNP data, and may be restricted to compute chromosome-specific GRMs for autosomes. The overall (or chromosome-specific) GRMs may then be analysed alongside phenotypic covariates to generate estimations of the variance explained by all SNPs included in the model. Because phenotypic measures were incomplete across individuals, and GCTA required phenotype measures on a per-individual basis; a subset was created of the variant file for each of the traits with only individuals that had a corresponding trait value as described above (*Genetic Sequencing Data, Variant Calling, and Filtering* section, Figure 1C).

We calculated overall heritability of the phenotypic axis using seven models, one for each phenotypic trait. We fitted the 29 autosomes of the starling genome to the seven models simultaneously, and all seven traits reached convergence. We estimated the proportion of variance explained by each chromosome, we ran each of the 29 autosomes (using the option—autosome to create the GRM) and Z chromosome (using the option—make-grm-xchr to create the GRM) individually, with one separate model of each phenotype trait. We calculated the 65% confidence interval (CI) for each trait (h_snp_—(1*S.E.)), and considered traits that had a lower 65% confidence interval above zero to be heritable according to this analysis.

For traits that were found to have a non-zero heritability, we estimated the relationship between the amount of explained genetic variance partitioned to each chromosome, and the chromosome size, we used a linear regression *lm*() in R. Chromosome sizes were calculated using Samtools v1.9 (Li et al., 2009). Additionally, we regressed chromosome size against per-chromosome gene number, as well as per-chromosome variant counts from the SNP data set used for the analysis.

### Genome-Phenotype Association Test (Aim 3)

To identify SNPs that were strongly associated with phenotypic traits (Figure 2: aim 3), BayPass v2.1 (Gautier, 2015) was used to perform GWAS against each of the seven phenotypic traits separately. We identified phenotypic associations by calculating the per-SNP Bayes factor (BF) under the standard covariate model (BF_is_) and the auxiliary covariate model (BF_mc_) to evaluate the association of SNP frequencies with phenotype. For each SNP, the BF is expressed in deciban units (dB 10 log_10_(BF_is/mc_)). Following Jeffrey’s rule, SNPs with a BF_is/mc_ ≥ 20 were retained as indicating a SNP under “decisive” selection. From this analysis we obtained seven separate lists of SNPs, capturing which genomic sites were associated with each of the seven phenotypic measures.

We sought to determine if genetic variation of each of these seven groups of phenotype-associated SNPs was associated with variation of environmental variables. We ran a genome-phenotype association test using the seven phenotype-associated SNP lists generated above, and the environmental variables used in *Environmental and Spatial Associations With Genome Wide Genetic Variation* section (for details see Supplementary Appendix S1: Genome-phenotype association test).

We also sought to determine if any of the identified phenotype-associated SNPs appeared to be under selection. A selection subset of the above phenotype-associated SNPs was created by retaining only the SNPs that were also flagged as highly divergent across sample sites by further baypass analysis. For this, we ran the core model to generate XtX statistic for all sample SNPs. We then calculated a significance threshold for candidate SNPs under divergent selection across sample sites through the generation of a pseudo-observed data (POD) of 20,000 SNPs, and a 1% empirical threshold was calculated for the observed XtX. Through core model and POD analysis, we identified which SNPs were divergent between sample sites (XtX>22.628) and were associated with phenotype (BF_is/mc_ ≥ 20). We generated a list of candidate genes under selection associated with each of the phenotypic traits using Bedtools *intersect* (Quinlan and Hall, 2010), using the *S. vulgaris* vAU1.0 annotation (Stuart et al., 2021a). The categorisation of a phenotype-associated SNP as under selection (pooled across all phenotypic traits) was assessed against SNP π (nucleotide diversity, obtained using vcftools—site-pi) using the *aov*() function in R, to see if there was any relationship occurrence of an correlated SNP under selection and the degree of site polymorphism across the population.

### Variance in Phenotype, Genetics, and Environment (Aim 4)

To examine if phenotypic variation was dependent on either genetic variation, environmental variation, distance from introduction site, or an interaction of some or all these factors (Figure 2: aim 4), we ran a linear mixed-effects model (details below). The analysis for this section was conducted in R v3.5.3 (R Core Team, 2017).

We calculated summaries of phenotypic variation as well as genetic variation, termed “phenotypic dispersion” and “genetic dispersion” respectively. These measures, obtained through an ordination approach (see Supplementary Appendix S2: Variance in phenotype, genetics, and environment methods; Supplementary Figure S3), captures the distance between an individual’s phenotype or genotype and the mean value of that data set for all individuals within a sample site. For sample sites with low phenotypic or genetic variability, individuals are close to the mean phenotype in PCoA space, and so would have a small phenotypic dispersion value. Sample sites with high phenotypic or genetic variability have more individuals with larger phenotypic dispersion values, because they are more dispersed from the sample site’s centroid in PCoA space.

To capture and summarise sample site environmental variability, we used environment variables obtained above (see *Environmental and Spatial Associations With Genome Wide Genetic Variation* section) that captured climate variability: bio3 (isothermality), bio4 (temperature seasonality), and bio15 (precipitation seasonality). Additionally, we included variation in normalised difference vegetation index (NDVI) values obtained for the same genetic sample sites in Stuart et al. (2021b). To account for collinearity across environmental variables, and also to reduce model terms, these four variables were reduced to two through PCA using the function *InDaPCA*() (Podani et al., 2021) (axis with eigenvalues >1 retained: env axis 1 (PC1) captures temperature variation and a component of precipitation variation, env axis 1 (PC2) captures ground cover variation and a component of precipitation variation, Supplementary Figure S4).

We calculated the sample collection site’s distance from nearest major introduction (D2INT) within each of the two genetic subpopulations (primary VIC introduction site as Victoria harbour, primary NSW introduction site as Sydney harbour (Higgins et al., 2006)) using geodist package (Padgham et al., 2021).

We constructed a linear mixed-effects model fitted using maximum likelihood to assess trends underlying phenotypic dispersion patterns using the R function *lmer*() in the package lme4 v1.1-26 (Bates et al., 2015). In the model we included the interaction between the main effects of: D2INT, genetic dispersion, env axis 1, env axis 2, with age and sex included as random effects. Genetic cluster was also included because a random effect as the two main genetic subclusters previously identified in Stuart et al. (2021b) were confirmed for this version of the genetic data (Figure 1D). We initially ran the model with main factors at the highest level of interaction (four), and this was iteratively reduced until an interaction effect reported a significant *p* value, leaving the final model with second level interaction effects.

## Results

### Environmental and Spatial Associations With Genetic Variation (Aim 1)

**FIGURE 3:**
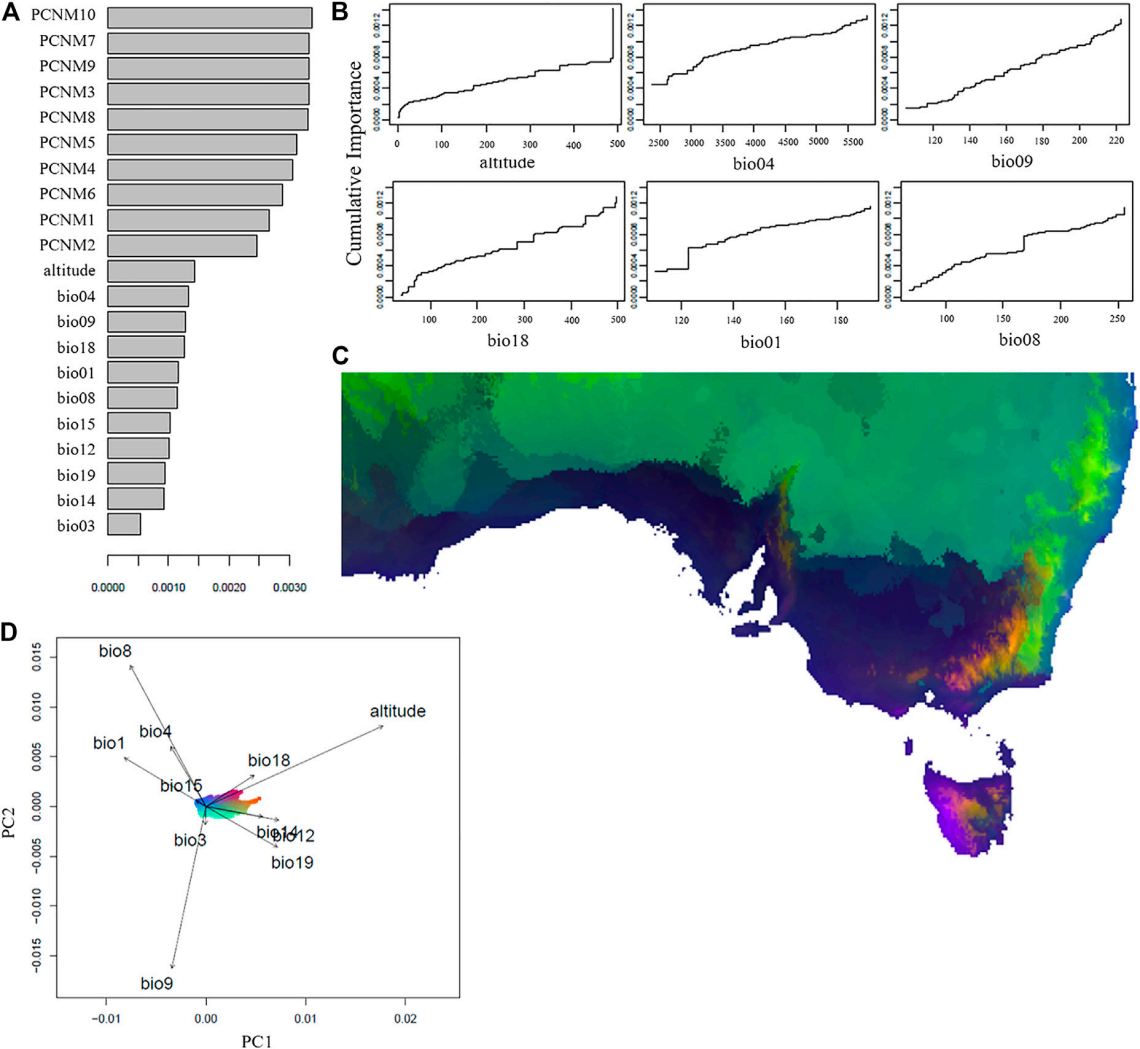
Environmental and spatial associations with the genetic variation across the Australian *Sturnus vulgaris* range, as analysed by GradientForest. Panel **(A)** depicts *R* ^2^ weighted importance of the environmental and spatial variables for explaining genetic gradients in the GF analysis. Panel **(B)** depicts the cumulative importance of allelic change along the six most important environmental gradients. Panel **(C)** depicts gradient forest prediction with similar colours representing similar expected genetic compositions based on the 11 assessed environmental variables, with panel **(D)** representing specific environmental predictors (represented by colour) and their relative importance. Environmental variables are bio1 (annual mean temperature), bio3 (isothermality), bio4 (temperature seasonality), bio8 (mean temperature of wettest quarter), bio9 (mean temperature of driest quarter), bio12 (annual precipitation), bio14 (precipitation of driest month), bio15 (precipitation seasonality), bio18 (precipitation of warmest quarter), bio19 (precipitation of coldest quarter), and altitude, while PCNMs represent axis of spatial patterns based on sample site latitude and longitudes.

The gradient forest (GF) analysis confirmed that the spatial variables each had a higher weighted importance in explaining genetic patterns than did any of the environmental variables considered. Of the environmental variables, the most important was altitude and bio04 (temperature seasonality), followed by bio09 (mean temperature of driest quarter), bio18 (precipitation of warmest quarter), bio1 (annual mean temperature), and bio08 (mean temperature of wettest quarter). Of the variables used in the analysis, the 10 spatial variables explained 81% and environmental variables explained 19% of the genetic variation (Figure 3A). There were a few major allelic compositional changes, indicated by a sharp jump in spline plots, most notably at 122 for bio1 and 170 for bio8 (Figure 3B), and some slight compositional change can be seen for bio18 from 280 to 500 units. Mapped projections of the GF predictions onto the environmental landscape of Australia identify high genetic differentiation due to different environmental variables along the eastern coastline, the northern coastline, inland along mountain ranges, and on the western half of Tasmania (Figure 3C). Along the eastern coastline, altitude, bio19, bio14, and bio12 appear to play the most important role in differentiating allelic patterns. Along the south coast of Australia bio8, as well as bio18, bio01, bio04, and bio15 are responsible for the genetic patterns in this region of the starlings’ Australian range (Figure 3D). There appears to be strong environmentally-associated differentiation between the lower and upper half of the eastern coastline, which resembles the patterns of genetic substructure identified previously (Figures 1A,D).

### Heritability and Chromosomal Partitioning of Phenotypic Variance (Aim 2)

We found that the overall SNP-based heritability values of the seven phenotypic traits was highly varied. Of the traits we measured, we found only two traits had h_snp_ 65% confidence interval that does not span 0; spleen mass (h_snp_ lower 65% CI = 0.311) and body mass (h_snp_ lower 65% CI = 0.193) (Table 1). The remainder of the assessed phenotypic traits had h_snp_ 65% lower confidence intervals spanning 0 (Table 1).

**TABLE 1:** SNP based heritability (h_snp_) values for *Sturnus vulgaris* phenotypic traits as calculated by GCTA. VP = phenotypic variance, V(G) = genetic variance, V(E) = environmental variance, h_snp_ = SNP-based heritability (Yang et al., 2017), S.E. = standard error. * Indicates a phenotype trait for which size corrected values were used in the heritability and chromosomal partitioning analysis. CI = confidence interval.

	VP	S.E.	V(G)	S.E.	V(E)	S.E.	h_snp_	S.E.	Lower 65% CI
Mass*	23.183	5.265	18.718	10.553	4.465	15.157	0.807	0.614	0.193
Tarsus	0.649	0.124	0.058	0.215	0.591	0.327	0.089	0.345	0
Head*	1.291	0.303	0.038	0.526	1.252	0.801	0.030	0.417	0
Beak*	35.944	7.547	0.000	13.377	35.944	20.227	<0.000	0.372	0
Wing*	14.956	2.125	0.000	3.002	14.956	4.739	<0.000	0.201	0
Spleen	0.002	0.001	0.002	0.001	<0.000	0.002	0.999	0.688	0.311
Heart	0.025	0.005	7.811e-03	0.010	0.017	0.015	0.318	0.459	0

Analysing heritability apportioned across chromosomes using GCAT for any traits found to have non-zero heritability estimates, we found that body mass had a significant linear relationship between the amount of variance explained by an individual chromosome and its respective length (F_1,28_ = 12.41, *p*-value = 0.001483, Adjusted *R* ^2^ = 0.2824) (Supplementary Table S2). We found that both per-chromosome gene count and SNP variant count was significantly associated with chromosome length (Supplementary Table S2).

**FIGURE 4:**
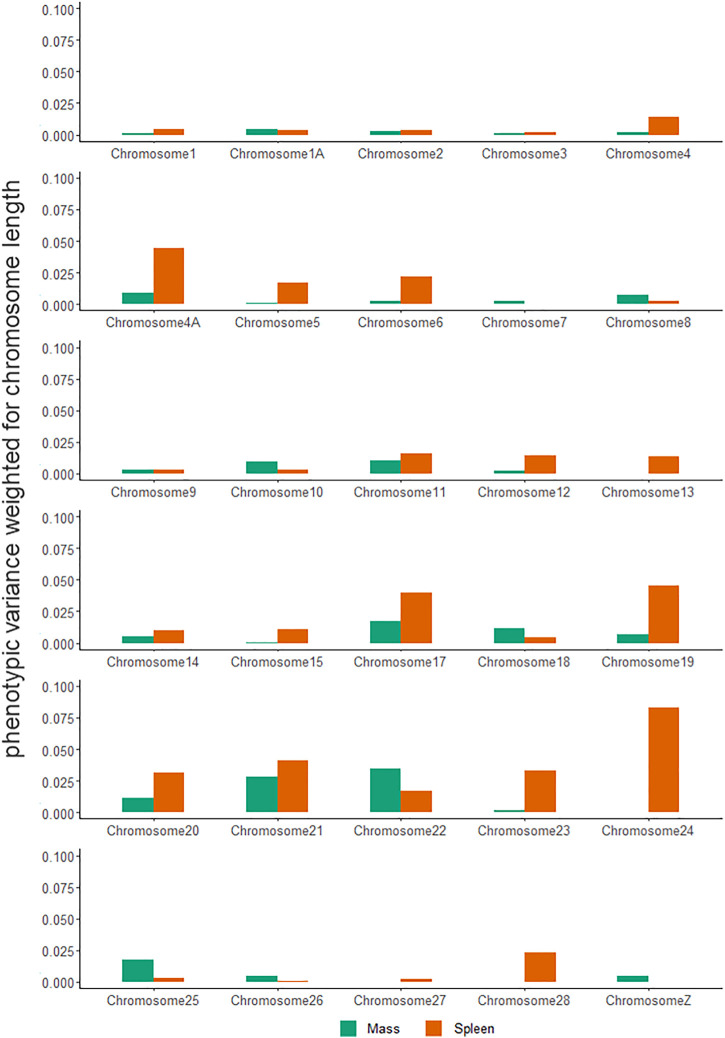
Weighted phenotypic variance explained by *Sturnus vulgaris* chromosomes across seven phenotypic traits. Values have been calculated by dividing the phenotypic variance estimated obtained by GCTA by chromosome lengths.

Chromosomal variance partitioning revealed that there was no single chromosome that explained a high amount of variance for all traits, with macrochromosomes (1–12), microchromosomes (13–27), and the sex chromosome (Z) explaining different amounts of the variance across the assessed traits (Figure 4, Supplementary Table S3). Generally, microchromosomes explained a large amount of phenotypic variance relative to their length, with body mass having the highest proportion of variance explained on chromosome 22, and spleen mass having the highest proportion of variance explained on chromosome 24.

### Genome-Phenotype Association Test (Aim 3)

We found that mass and beak surface area had the smallest number of correlated SNPs identified, while head antero-posterior cross section, as well as spleen and heart mass, had several hundred correlated loci across the two models (Table 2). We found moderate overlap in the phenotype-associated loci identified by the auxiliary covariate model and the IS covariate model in Baypass, with the latter model detecting the fewest number of loci (Table 2, Supplementary Figure S5).

**TABLE 2:** Number of SNPs correlated with phenotypic traits in *Sturnus vulgaris* as identified as being outliers possibly under selection by Baypass using the Auxiliary covariate model and the IS covariate model, and those identified as being under selection using the core model/POD analysis. Total phenotype-associated SNPs is the union of the SNP lists generated using the Auxiliary covariate model and the IS covariate model. Total phenotype-associated SNPs under selection is a subset of the row above, listing only those SNPs that were flagged as under selection by the baypass core model, with an XtX threshold of 22.628 determined by POD calibration.

	Mass	Tarsus	Head	Beak	Wing	Spleen	Heart
Auxiliary covariate model	54	88	430	53	113	294	259
IS covariate model	16	19	21	8	17	26	25
Total phenotype-associated SNPs	62	100	442	60	122	303	268
Total phenotype-associated SNPs under selection (XtX>22.628)	3	2	1	0	6	2	4

**FIGURE 5:**
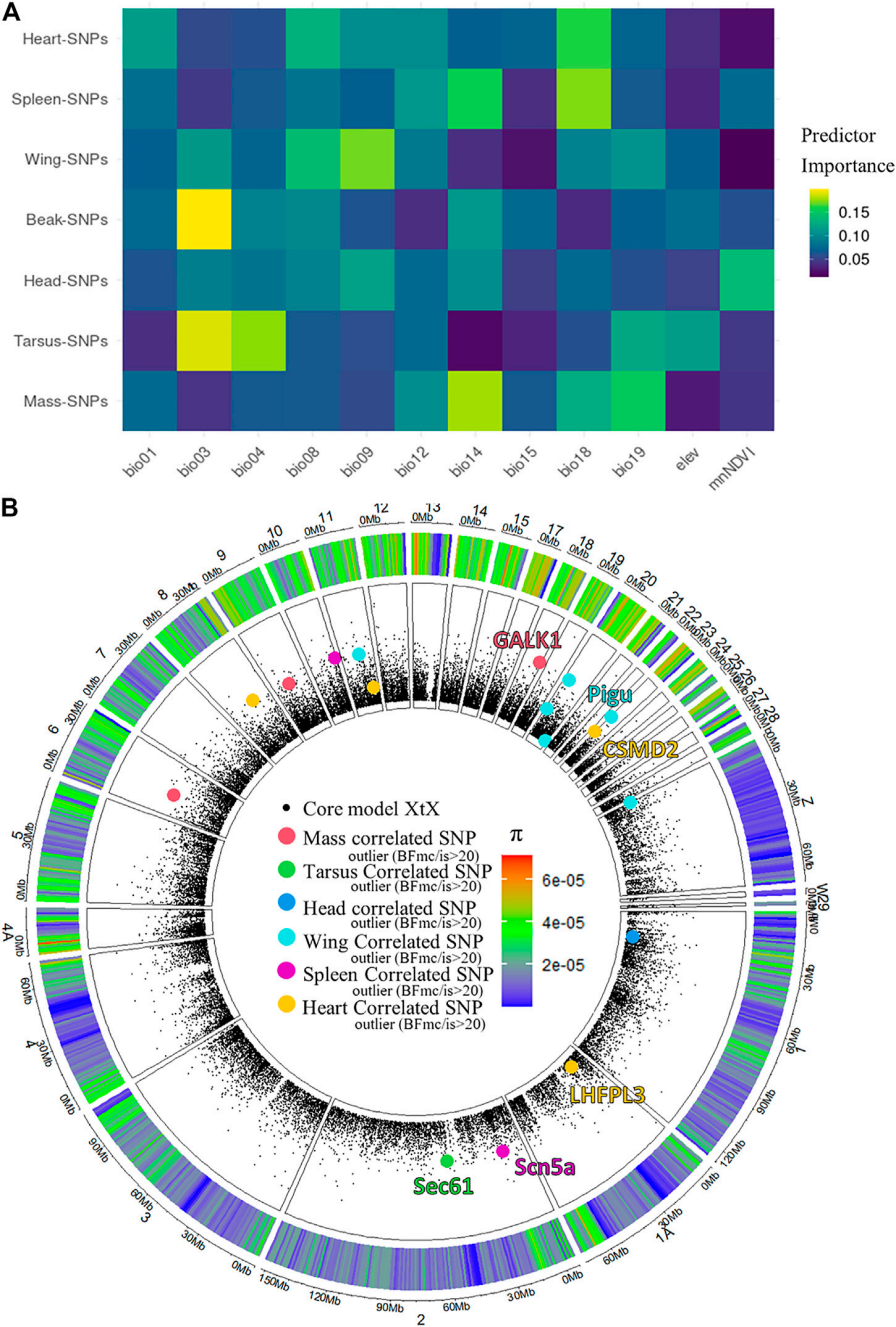
Phenotype-Genetic correlations, and environmental predictors across the Australian *Sturnus vulgaris* range. Panel **(A)** depicts a heatmap of relative environmental predictor importance on groups of phenotype-associated SNPs, with colour indicating the relative importance of each environmental variable in explaining PCA-summarised genetic information (rows add up to unity) with environmental predictors bio01 = Annual Mean Temperature, bio03 = Isothermality, BIO4 = Temperature Seasonality, BIO8 = Mean Temperature of Wettest Quarter, bio09 = Mean Temperature of Driest Quarter, bio12 = Annual Precipitation, bio14 = Precipitation of Driest Month, bio15 = Precipitation Seasonality (Coefficient of Variation), BIO18 = Precipitation of Warmest Quarter, BIO19 = Precipitation of Coldest Quarter, elev = altitude, mnNDVI = mean normalized difference vegetation index. Panel **(B)** depicts all phenotype-associated SNPs under selection across the starling genome, with overlapping genes annotated.

We performed a genome-phenotype association test and found that across the seven phenotype-associated groups of SNPs, a variety of different environmental predictors explained patterns of genetic variance (Figure 5A). The environmental predictor reporting the highest relative importance for any phenotypic trait was bio03 (isothermality) on beak-correlated SNPs. Considering the relative importance of environmental predictors within each phenotypic variable, it appeared that wing, beak and tarsus were most highly associated with temperature variables (bio01-bio09), while body mass, spleen mass, and heart mass were most highly associated with precipitation variables (bio12-bio19). Some environmental variables, such as bio01 (annual mean temperature), bio08 (mean temperature of wettest quarter), bio12 (annual precipitation), bio15 (precipitation seasonality), and elevation, did not appear to significantly correlate with any tested phenotypic variable. The amount of genetic variation explained by the environmental variables varied from approximately 1–2% across the seven phenotype-associated groups of SNPs (Supplementary Figures S6, S7).

Only a small fraction of phenotype-associated SNPs were flagged as being under selection across the starling’s Australian range using BayPass (0–5%, Table 2), and we identified a small number of the genes to which these sites mapped (Figure 5B). These included mass-associated galactokinase 1 (*GALK1*), tarsus-associated Protein transport protein SEC61 (Sec*61*), wing-associated Phosphatidylinositol glycan anchor biosynthesis class U protein (*PIGU*), spleen-associated Sodium channel protein type 5 subunit alpha (*Scn5a*), and heart-associated LHFPL tetraspan subfamily member 3 protein (*LHFPL3*) and CUB and sushi domain-containing protein 2 (*CSMD2*) (Figure 5B, Supplementary Table S4). We did not find any relationship between a SNP site being categorised as BayPass outlier or not Baypass outliers and SNP π (F_8,128,208_ = 1.802, *p*-value = 0.0716) (Figure 5A).

### Variance in Phenotype, Genetics, and Environment (Aim 4)

We found that phenotypic dispersion, averaged within sample sites, was significantly correlated with Hs values previously calculated to capture the within local gene diversity (F_1,12_ = 12.7, *p*-value = 0.00389, Adjusted *R* ^2^ = 0.4738) (Supplementary Figure S3).

**FIGURE 6:**
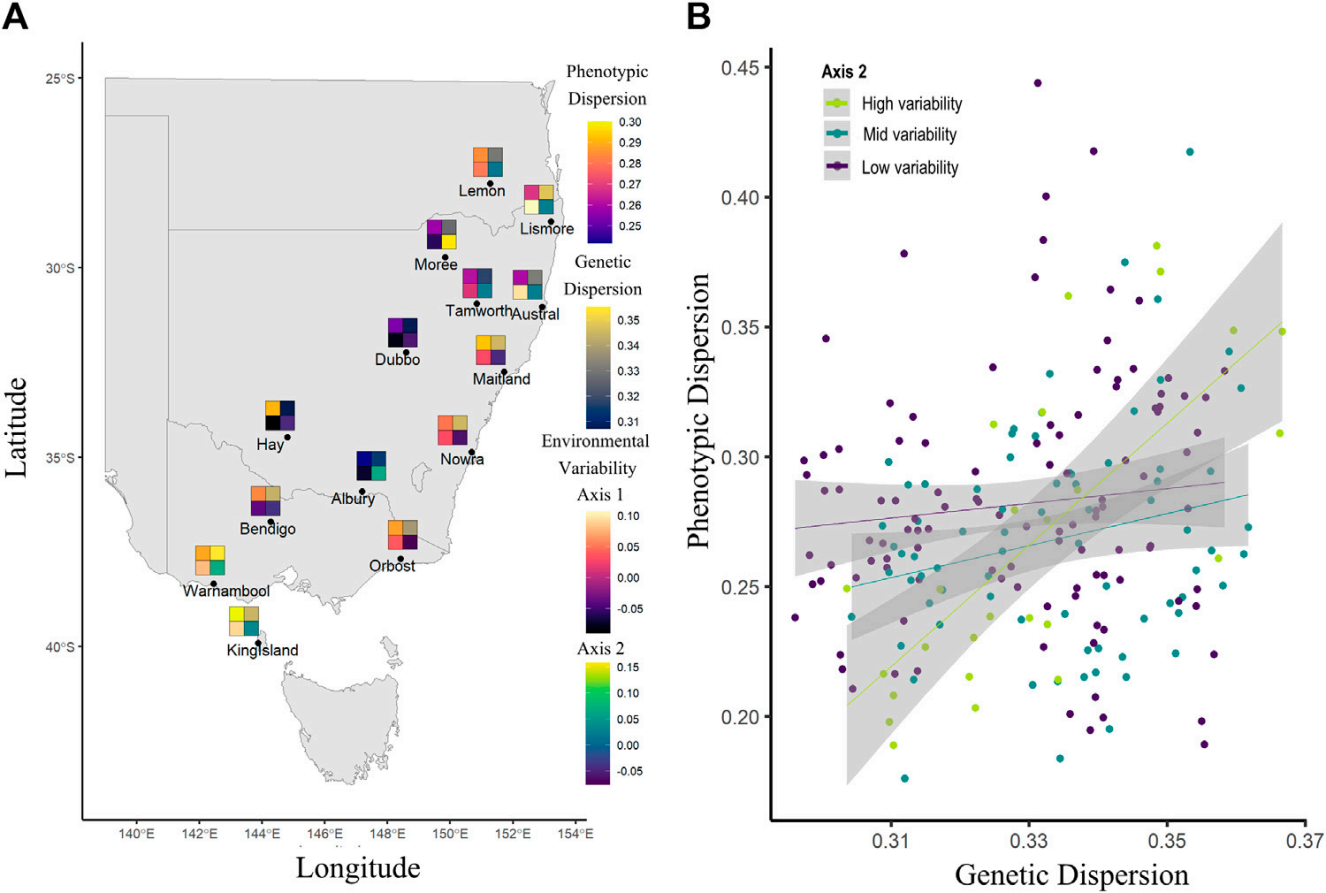
Trends in *Sturnus vulgaris* phenotypic, genetic, and environmental variability, with panel **(A)** average sample site measures for phenotypic dispersion (upper left), genetic dispersion (upper right), environmental axis 1 (temperature variation and a component of precipitation variation) (lower left), and environmental axis 2 (ground cover variation and a component of precipitation variation) (lower right), and panel **(B)** representing the significant interaction between env axis2 and genetic dispersion on phenotypic dispersion.

Using our linear mixed effect model to assess the effects of genetic and environmental variance on phenotypic variance, we found only one significant second level interaction, which was the interaction of genetic dispersion and env axis 2 (ground cover variation and partially annual precipitation variation) (Table 3; Figure 6). This interaction showed that when ground cover variation and precipitation variation is low, there is little change in phenotypic dispersion, regardless of the underlying level of genetic diversity. However, when there is high variability in vegetation cover and precipitation, we see a decrease in phenotypic dispersion when genetic dispersion is low, and an increase in phenotypic dispersion when genetic dispersion is high (Figure 6B). More generally, we identified a significant correlation between genetic dispersion and phenotypic dispersion but found no significant effect or interaction effect with D2INT or env axis 1 (Table 3).

**TABLE 3:** Factors affecting phenotypic dispersion in *Sturnus vulgaris,* using a linear mixed effect model examining correlation to distance to introduction site (D2INT), genetic dispersion, env axis 1 (temperature variation and a component of precipitation variation), and env axis 2 (ground cover variation and a component of precipitation variation).

Fixed effects	Std. Error	Df	t value	Pr (>|t|)	Sig
(Intercept)	6.72E-03	1.69E+00	41.696	0.00152	**
D2INT	3.27E-03	2.09E+02	−0.939	0.34858	
Env Axis 1	4.59E-03	1.47E+02	0.101	0.91983	
Env Axis 2	4.02E-03	2.08E+02	−1.409	0.16045	
Genetic dispersion	4.55E-03	2.02E+02	2.138	0.03368	*
D2INT * Env Axis 1	4.76E-03	2.09E+02	−0.685	0.4942	
D2INT * Env Axis 2	3.79E-03	2.08E+02	0.178	0.85923	
D2INT * Genetic dispersion	4.47E-03	2.09E+02	0.161	0.87244	
Env Axis 1 * Env Axis 2	5.95E-03	2.05E+02	−0.606	0.54537	
Env Axis 1* Genetic dispersion	4.01E-03	2.11E+02	−1.128	0.2608	
Env Axis 2 * Genetic dispersion	5.21E-03	2.07E+02	2.665	0.00829	**

## Discussion

Our results demonstrate that environmental variables (particularly elevation) play important roles in shaping spatial trends Australian starling genetics. The relationship between allelic frequencies and environmental variables across the starlings’ range may be indicative that strong spatial patterns associated with historic introductory regimes could be reinforced by differing environmental gradients during colonisation and expansion. Further, we identified that both genetics and plasticity explain previously identified ecogeographical phenotype trends across the Australian range. We examined a range of phenotypic traits that appear to be heritable (body mass and spleen mass) or negligibly heritable (e.g. beak surface area and wing length) across the starlings’ Australian range. For SNP variants associated with each of these phenotypes, we identify key environmental variables that correlate with genetic patterns, identifying temperature and precipitation variables that putatively play important roles shaping phenotype in this invasive population. Finally, we illustrate that overall phenotypic dispersion is correlated with underlying genetic variability, and that these interact positively with the level of vegetation variation within a region, suggesting that an interaction between ground cover and genetic variation plays an important role in shaping plastic development in Australian starlings.

### Environmental Predictors of Allelic Patterns Across the Australian Range

Examining environmental and spatial predictors of *S. vulgaris* allelic patterns across the Australian range, it is unsurprising that the spatial variables explain such a large proportion of the overall trends. This confirms previous isolation-by-distance analysis that identifies physical distance between sites as the largest determinant of genetic patterns (Stuart et al., 2021b). However, the gradient forest (GF) analysis does allow us to identify underlying environmental associations independent of spatial effects.

The environmental variables of altitude and temperature seasonality are identified as the two most important predictors of allelic change across the Australian range (Figure 3A). The importance of altitude is surprising, because starlings have an upper elevation limit of 2000m (Higgins et al., 2006), and because the maximum altitude of the sampling sites was only 500–600 m. Nevertheless, we see elevation having a strong effect on allelic frequencies at this upper most sampling limit, with the step plot depicting a sharp rise in importance of altitude at around 500 m (Figure 3B). Selection mediated by elevation may be a result of direct selection pressure via partial oxygen pressure shifts (Lim et al., 2021), or due to co-varying factors such as ecosystem assemblage differences (e.g. Samuel et al., 2015). Some of these differentiating patterns follow the Great Dividing Range, a band of mountains that stretches along most of the eastern Australian coastline forming the continental divide. The Great Dividing Range plays a variable role in limiting avian geneflow (Pavlova et al., 2013; Keighley et al., 2020), and though no strong population difference is seen across this range in Australian starlings (Stuart et al., 2021b), our results suggest elevation is likely still exerting small scale allelic effects. The second most important environmental predictor was temperature seasonality, providing further evidence that temperature fluctuations, which also captures extreme temperature events, may be a more relevant driver of survival and evolution of small insectivorous avian species, compared to mean temperatures (Vasseur et al., 2014; Gardner et al., 2017).

Region-specific patterns indicate that a relationship between both higher temperatures and higher precipitation plays an important role in shaping allelic trends along the southern Australian coastline (Figures 3C,D). Meanwhile, allelic patterns along the eastern Australian coastline appear to covary primarily with altitude- and precipitation-related variables.

Even when removing spatial effects on allele frequencies through GF analysis, a divide between the south and east of the starlings’ range is evident. It is possible that differing selection regimes imposed in the climatically different introduction points meant that during colonisation and expansion in these two subpopulations they became locally adapted within their regions. Each subpopulation now remains distinctive, not just because of neutral allele patterns such as genetic drift occurring independently within the subpopulation, but because environmental variables have shaped genetic variants differentially. Finally, for inland Australia the mean temperature of the driest quarter is most important for explaining patterns of allelic diversity, likely due to radical drops in precipitation, and this increases with distance from the continental divide. These results align with predicted environmental PCA based sample site groupings identified in Stuart et al. (2021b), and identify which of the differential climate variables are having the greatest effect within a region.

### Heritability and Non-heritable Plasticity Across Phenotypic Traits in an Invasion

We report a wide range of heritability values across the seven phenotypic measurements assessed as part of this study. The large error bars resulting from the small study sample size does mean that cross-study comparisons of these heritability values are less useful (particularly true for lower heritability values; Visscher et al., 2014). However, using the 65% CI around the estimated h_snp_ values, we identify that the phenotypic measures of body and spleen mass had snp-based heritability values above 0, and presumably higher than the snp-based heritability values of the remaining phenotypic traits with h_snp_ measures not significantly different from 0.

While we interpret the heritability values for body and spleen mass as indicating these phenotypic traits are like more heritable than the others used in this study a true heritability value approaching unity (as has been estimated by the h_snp_ values) would be biologically unlikely, and thus we may expect the true h_snp_ values to be towards the lower end of the confidence interval. Nevertheless, it is apparent that trends in body and spleen mass across Australia appear to be correlated with genetic relatedness, an interesting result considering that both these traits may be strongly influenced by non-genetic factors (Smith and Hunt, 2004; Husby et al., 2011) alongside heritable components (Ubosi et al., 1985; Rønning et al., 2007). It is possible that selection during range expansion in response to novel pressures have resulted in selection and high heritability estimates for these traits, such as summer maximums driving mass clinal variation (Andrew et al., 2018), or effects of infection, and parasite exposure/release on an invasive species’ spleen mass (Ardia, 2005; Brown et al., 2015; Martin et al., 2015). The limitations of correlation-based heritability estimates must be acknowledged here however, as correlation analysis discern if positive associations are causal in nature, and so these trends may in fact be driven by an external adaptive or demographic factor. The remainder of the phenotypic traits assessed as part of this study were identified as having negligible heritability estimates: beak surface area, wing length, tarsus length, head antero-posterior cross section, and heart mass. Such low heritability is not unheard of, as similarly low heritability estimates have been reported in other species, for instance Silva et al. (2017) found zero wing length heritability estimated for the house sparrow (*Passer domesticus*) (Silva et al., 2017). Likely then these traits are determined more so by plastic responses, though it is possible that the correlation-based analysis resulted in an underestimation of heritability in our data. Underestimation would likely be the case if phenotypic variation is determined by a very small number or singular SNP variants, that act independently to the overall genetic similarity used for heritability estimation in GCTA (Domingue et al., 2016). Hence even for traits found to have a h_snp_ estimate non-significantly different from zero we still perform phenotype-association and outlier detection tests using baypass.

Out of all the phenotypic traits assessed in this study, only the degree of variance in body mass was found to be heritable and significantly associated with chromosome size (Figure 4A). Body mass is a complex and undoubtedly highly polygenic trait, and has been found to behave similarly across many different species and studies (Robinson et al., 2013; Bérénos et al., 2015; Santure et al., 2015; Silva et al., 2017), and appears to behaving likewise within this invasive population.

Interpreting these trends broadly, we infer that body mass and spleen mass are more dependent on underlying genetics, while phenotypic traits with non-significant h_snp_ values such as beak surface may vary only with environmental gradients, irrespective of broad underlying genetics. Considering these results within the context of previous findings in this species (Cardilini et al., 2016), the ecogeographical relationships identified in Australian starlings in that study may result from genetic gradients or environmentally driven gradients. Maximal temperatures predicted patterns of variation in body mass and bill surface area (Cardilini et al., 2016), consistent with Bergmann’s (Meiri and Dayan, 2003) and Allen’s (Allen, 1877) rules, which predicts small body size and larger bill size in warmer climates. We may then infer that while smaller body size in response to higher temperatures are likely to be more broadly heritable, patterns in beak surface area likely arise due to plastic effects, at least within the Australian starling population. Such hypothesis may be tested in future common garden experiments that raise individuals under standardised conditions, with additional focus on regions with differing frequencies of key phenotype-associated SNPs.

### Phenotypic, Genetic, and Environment Correlates

The above two sections have demonstrated that despite strong spatial genetic patterns, environmental variables are shaping allelic trends across Australian starlings, and that phenotypic traits across the Australian range have different levels of heritability. Following on from this, we examined relationships between phenotypic, genetic, and environmental variability, and assessed which environmental variables appeared to be shaping individual phenotypic traits.

Unsurprisingly, our study found that within Australian starlings, overall phenotypic variance is correlated with genetic variance, but the strength of this relationship is primarily influenced by precipitation and vegetative ground cover variation, rather than variation in temperature. This result suggests that stable temporal patterns of ecosystem plant assemblages help to maintain less variable phenotypes, presumably through plasticity because phenotype does not depend on genotype in this circumstance. Conversely, highly variable ground cover may interrupt plastic convergence to this general-purpose phenotype, so that genetics assumes more importance. Determinants of phenotypic plasticity within populations has a major impact on long term avian species persistence (Vedder et al., 2013). Our results demonstrate that environmental stability may be a key consideration in limiting or maintaining plasticity in this species. Because increases in environmental variability are associated with climate change (Thornton et al., 2014), we must consider that the evolved plasticity limits of species may be reached, impeding their ability to respond rapidly to change and thereby endangering long term persistence of populations (Reed et al., 2010).

With respect to trait-specific environmental predictors, we found the strongest association between isothermality (daily verses seasonal temperature oscillations) and beak surface area-associated SNPs. Previous research using maximal temperature data concluded that beak surface measurements adhere to Allen’s rule (Allen, 1877; Cardilini et al., 2016), reflecting a widely found pattern of shape shifting in response to warmer environments (Ryding et al., 2021). Our results suggest that the trend may be more so driven by patterns of temperature variability. We also identified high importance of temperature variability measures in explaining tarsus-associated SNP genetic patterns. A non-significant association between temperature and this phenotypic measure was found previously (Cardilini et al., 2016), however only temperature extremes were tested in these initial models (maximum summer temperature and minimum winter temperature). It is possible then that assessment of phenotypic adherence to Allen’s rule may sometimes need to be expanded to cover temperature variability.

In agreement with previous findings regarding heart mass not adhering to Hesse’s rule (organisms will have larger hearts in colder environments) (Cardilini et al., 2016), we see that precipitation has the greatest effect on heart mass-associated SNPs, particularly precipitation of coldest quarter. Precipitation also strongly correlates with genetic patterns in spleen mass and body mass-associated SNPs. A significant correlation has been found between body condition and spleen sizes, and climatic conditions (including precipitation) across numerous avian species, with the direction of effects mediated by the nature of parasite-induced natural selection within the species (Møller and Erritzøe, 2003). Considering that both spleen mass and body mass were found to be heritable, we may hypothesise that precipitation during warm and dry months may have a strong effect on fitness, and hence adaptation, within this population. An alternate explanation for these genetic, environmental, and phenotypic correlations is that these patterns have resulted from range expansion along environmental gradients. Therefore, future analyses of these traits should investigate whether range edge effects can be detected in genes of interest. Four phenotype-associated genes (*GALK1, Scn5a, LHFPL3,* and *CSMD2*) were flagged as under selection across these three traits and may play important adaptive roles in responding to precipitation regimes across Australia, and other environmental factors that may covary with precipitation (e.g. via *CSMD2* mediated pathogen responses (Alber et al., 2020)). Despite the importance of elevation in shaping broad allelic patterns across the Australian starlings’ range, it does not appear to play a particularly important role in influencing any of the phenotypic traits measured as part of this study, or at the very least does not appear to co-vary with phenotype-associated SNPs identified in this study.

## Conclusion

Within the novel invasive Australian range, we found that adherence to biologically significant ecogeographical patterns within starlings may be accomplished through a complex sum of heritable and non-heritable components. Heritable components of selection may be driven by only a small number of biologically significant SNPs, for which oftentimes temperature variability or precipitation during warmer, dryer months play a vital role. A better understanding of environmental and phenotypic interaction is invaluable for helping predict climate shift responses (Tian and Benton, 2020). Further, to what extent phenotypic shifts are non-heritably plastic gives us important information about how climate shifts may result in lost genetic diversity. Functional restrictions in avian genetics (O’Connor et al., 2019) mean that selection driven by ecogeographical patterns in many species may result in allelic diversity loss for less plastic traits. Investigating phenotypic shifts within invasive populations, particularly those that differ to the species’ native climate, provide an indirect means of assessing climate change vulnerability.

## Data Availability

This study is based on previously published data (Stuart et al., 2021a), the raw versions of which can be found under NCBI BioProject accession PRJNA655259. Data processing and analysis code is available on GitHub (https://github.com/katarinastuart/Sv6_StarlingEcogeographical).
