# Supplementary material for: Genetics and Plasticity Are Responsible for Ecogeographical Patterns in a Recent Invasion
Source: Front Genet. 2022 Mar 11;13:824424. doi: 10.3389/fgene.2022.824424 (PMC8963341; doi:10.3389/fgene.2022.824424)
Supplement: Supplementary file 1 [file DataSheet1.docx]

**Genetics and plasticity are responsible for climate induced ecogeographical patterns in a recent invasion**

Katarina C. Stuart^1^*, William B. Sherwin^1^, Adam P.A. Cardilini^2^, Lee A. Rollins^1^

^1^ Evolution & Ecology Research Centre, School of Biological, Earth and Environmental Sciences, UNSW Sydney, Sydney, New South Wales, Australia

^2^ School of Life and Environmental Sciences, Deakin University, Waurn Ponds, VIC, Australia

* Corresponding Author: Katarina Stuart (Katarina.Stuart@unsw.edu.au)

**Supplementary Materials**

**Appendix 1: Genome-phenotype association test methods**

We used the phenotype-associated SNP lists produce seven variant files subset from the original ‘phenotype analysis’ variant file, that were each summarised using PCA using plink v1.9 and the first 20 axes retained. We ran a separate step linear model for each group of SNPs using the *lm()* function in R, with the genetic PCA axis eigenvalues serving as the response variable, and a selection of environmental variables serving as the predictors. Environmental variables used were those obtained during the GF analysis (see main manuscript, section 2.3. Environmental and spatial associations with genome wide genetic variation). A separate linear model was run for each of the 20 PCA axes eigenvalues, and the overall model R^2^ values (amount of genetic PCA axis variance explained by environmental predictors) were weighted by the percentage variance each of the genetic axis captured of the overall variance across all genetic PCA axis. *A priori*, we decided to plot weighted variance explained for all models and retain only the axis above the plateau (Supplementary Materials: Fig. S6). This decision was made because PCA of genetic SNP data often contain many axes that each capture small components of background variation in the genetic data set, however we were interested in assessing only the major patterns of variation in the data set that appeared to be influenced by environment. Across the seven phenotypic measures, this left us with one genetic PCA axis for four traits (mass, tarsus, beak, wing), two genetic PCA axes for spleen, and four genetic PCA axes for head and heart (Supplementary Materials: Fig. S7). Once the total number of major genetic PCA axis was determined, the relative importance of environmental predictors on genetic PCA axis loadings was determined using the *calc.relimp()* function in the relaimpo package in R. For phenotype-associated SNP groups that reported more than one genetic PCA axis having a strong correlation with environment (i.e. spleen, head, and heart), the relative importance of the environmental predictors were weighted relative to the variance of each of the genetic PCA axis, such that the total relative importance of each environmental predictors on a phenotype-associated SNP group totalled to unity.

**Appendix 2: Variance in phenotype, genetics, and environment methods**

We calculated a summary of phenotypic measure variation called ‘phenotypic dispersion’, from the seven phenotypic traits. Phenotypic dispersion captures the distance between an individual’s phenotype and the mean value of the trait for all samples, and is calculated using an ordination approach (Guillerme et al., 2020). We scaled each sample site’s phenotypic data using the *scale()* function, and then processed the data using the gowdis() function, then the *betadisper()* function in the package Vegan (Oksanen et al., 2019). The function gowdis measures dissimilarity of data sets of mixed variables, while the function betadisper is used to compute the average distance of objects within a dissimilarity matrix in Principal Coordinates Analysis (PCoA) space. We retained the distance of each individual to the centroid of that individual’s sampling site (the centre of mass of the geometric object formed by the points in PCoA space).

We used a similar approach to extract ‘genetic dispersion.’ We calculated a pairwise genetic distance matrix of the 212 individuals using the stamppNeisD() in the R package StAMPP v1.6.1 (Pembleton et al., 2013). We ran this distance matrix through the betadisper() function and an individual’s distance to their sample site’s mean genotype was calculated in PCoA space, to be interpreted as above. To validate this as a measure of local genetic diversity, we regressed the sample site averages of genetic dispersion against Hs (the within population gene diversity) calculated for the sample sites in Stuart & Cardilini (2020) (Supplementary materials: Fig. S3).


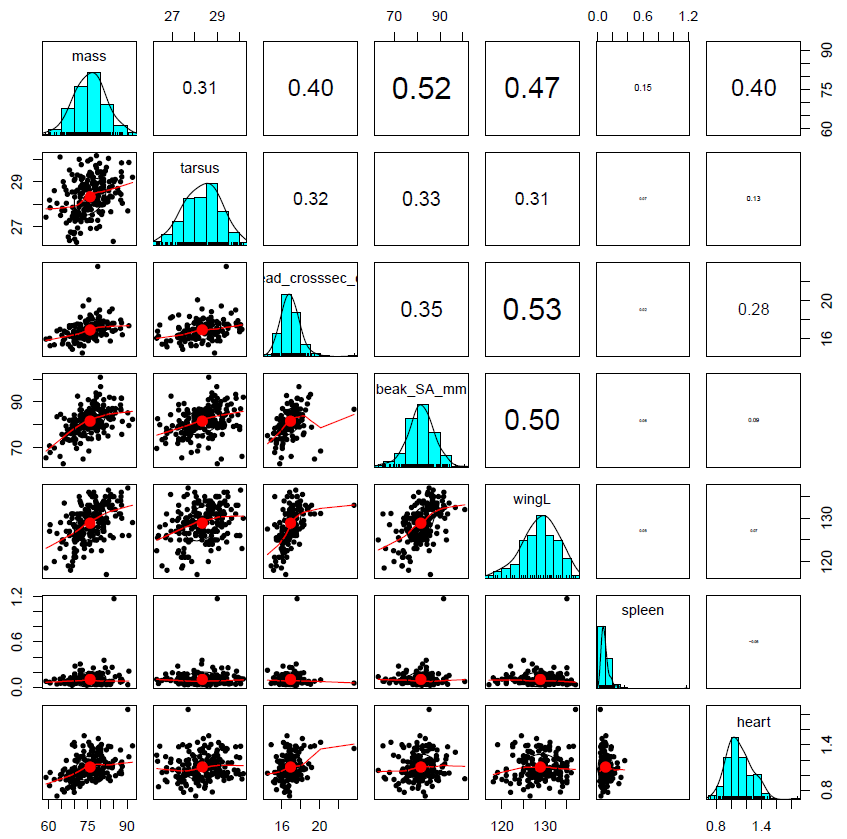


**Figure S1:** **Phenotype correlations** across all 212 individuals using the *pairs.panel()* function in psych.

**Table S1: Correlation between Tarsus and phenotypic traits in *Sturnus vulgaris*** measures across 14 populations.

|  | **Sample Size** | **Adjusted R-squared** | **F-statistic** | **DF** | **P value** | **Size corrected** |
| --- | --- | --- | --- | --- | --- | --- |
| **mass** | 212 | 0.09421 | 22.95 | 1,210 | 3.147e-06 | yes |
| **head antero-posterior cross section** | 143 | 0.09538 | 15.97 | 1,141 | 0.0001032 | yes |
| **beak surface area** | 199 | 0.1067 | 24.66 | 1,197 | 1.478e-06 | yes |
| **wing length** | 201 | 0.08943 | 20.64 | 1,199 | 9.589e-06 | yes |
| **spleen** | 179 | -0.001418 | 0.7494 | 1,176 | 0.3878 | no |
| **heart** | 184 | 0.01106 | 3.046 | 1,182 | 0.08263 | no |


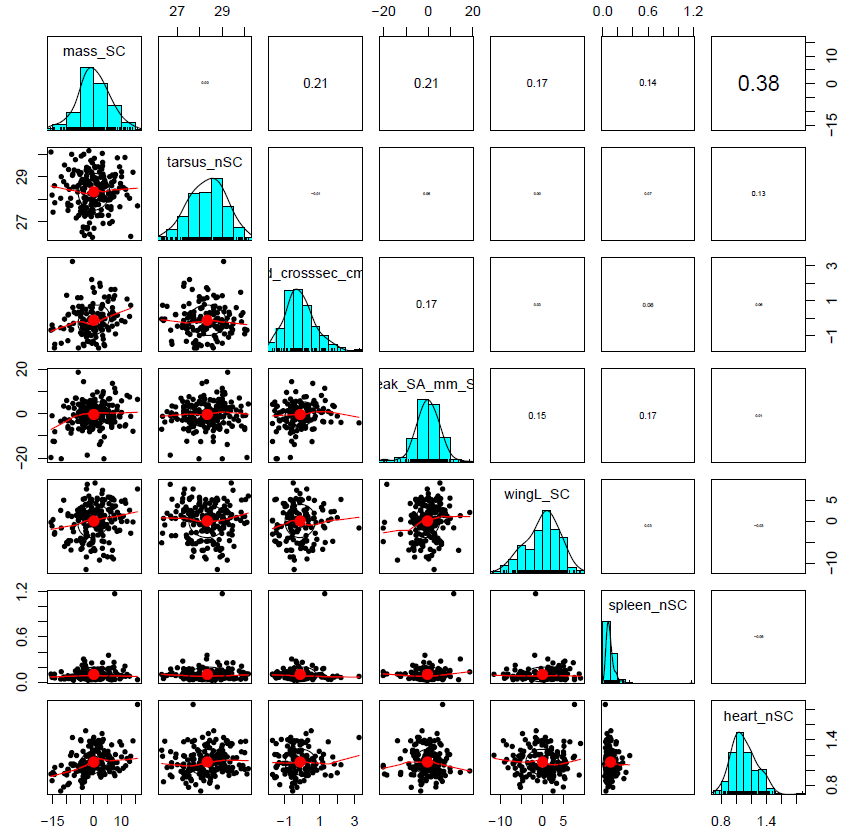


**Figure S2:** **Size corrected phenotype correlations** across all 212 individuals using the *pairs.panel()* function in psych.


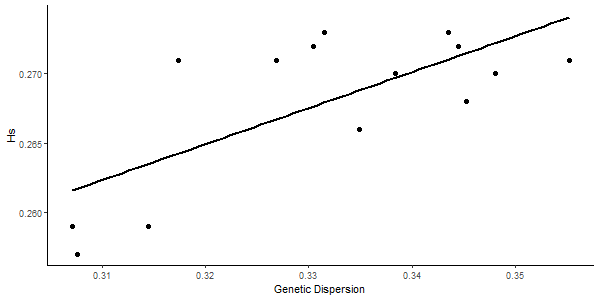


**Figure S3:** Regression of Hs and genetic dispersion


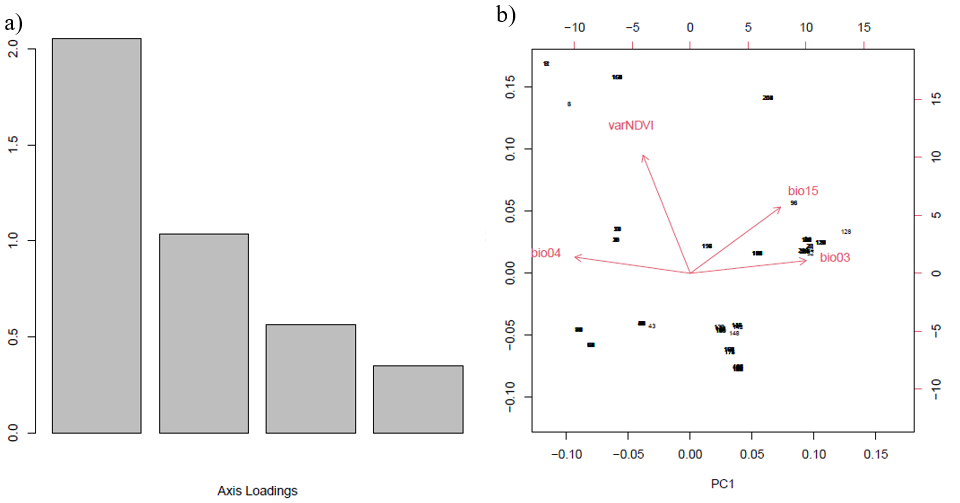


**Figure S4: InDaPCA of environmental variables**, panel a) PCA axis eigenvalues, and panel b) biplot.

**Table S2: Chromosome partitioning regression analysis for *Sturnus vulgaris* SNP based heritability** across seven phenotypic traits as determined by proportion variance values calculated with GCTA.

| **Chromosome size vs** | **F-statistic** | **DF** | **P-value** | **Adjusted R-squared** |
| --- | --- | --- | --- | --- |
| Total genes per chromosome | 745.40 | 1, 30 | <0.000 | 0.96 |
| Total variants per chromosome | 219.00 | 1, 30 | <0.000 | 0.8755 |
| Body Mass | 12.41 | 1,28 | 0.001 | 0.2824 |
| Tarsus Length | 0.54 | 1,28 | 0.470 |  |
| head antero-posterior cross section | 0.03 | 1,28 | 0.858 |  |
| beak surface area | 3.53 | 1,28 | 0.071 |  |
| wing length | 0.86 | 1,28 | 0.362 |  |
| spleen mass | 3.33 | 1,28 | 0.079 |  |
| heart mass | 1.45 | 1,28 | 0.239 |  |

**Table S3: Proportion of phenotypic variance (h^2^) explained by each *Sturnus vulgaris* chromosome** across the seven phenotypic traits.

| Chr | SNPs | Size (Mb) | Mass | | Tarsus | | | Head | | |
| --- | --- | --- | --- | --- | --- | --- | --- | --- | --- | --- |
|  |  |  | *V(G)/V(P)* | SE | *V(G)/V(P)* | SE | *V(G)/V(P)* | | SE |  |
| All |  | 1029.606 | 0.807406 | 0.614011 | 0.089394 | 0.345429 | 0.029709 | | 0.413611 |  |
| 2 | 11146 | 126.122 | 0.176835 | 0.169732 | 0.382652 | 0.246148 | 0.019919 | | 0.245237 |  |
| 3 | 6559 | 73.37754 | 0.354115 | 0.2197 | 0.000001 | 0.16347 | 0.000001 | | 0.211797 |  |
| 4 | 12692 | 151.9278 | 0.406237 | 0.249965 | 0.000001 | 0.180839 | 0.197378 | | 0.306595 |  |
| 5 | 10250 | 107.3521 | 0.149775 | 0.190749 | 0.000001 | 0.147926 | 0.000001 | | 0.232305 |  |
| 6 | 6577 | 72.52561 | 0.157888 | 0.167409 | 0.000001 | 0.144064 | 0.205562 | | 0.230519 |  |
| 7 | 3685 | 22.39173 | 0.201558 | 0.148306 | 0.060607 | 0.12269 | 0.13957 | | 0.18063 |  |
| 8 | 7676 | 58.5824 | 0.058422 | 0.159682 | 0.025409 | 0.147883 | 0.000001 | | 0.242286 |  |
| 9 | 4633 | 35.15055 | 0.098828 | 0.125976 | 0.000001 | 0.117077 | 0.000001 | | 0.175636 |  |
| 10 | 4528 | 38.36043 | 0.106623 | 0.13781 | 0.150248 | 0.138152 | 0.332714 | | 0.255797 |  |
| 11 | 4688 | 31.01029 | 0.239202 | 0.166184 | 0.000001 | 0.115274 | 0.000001 | | 0.15924 |  |
| 12 | 4827 | 25.75342 | 0.079992 | 0.115815 | 0.000001 | 0.115159 | 0.000001 | | 0.172495 |  |
| 13 | 3034 | 19.02258 | 0.174843 | 0.11546 | 0.032748 | 0.10742 | 0.257183 | | 0.211843 |  |
| 14 | 3928 | 22.3777 | 0.235064 | 0.177565 | 0.137881 | 0.14472 | 0.000001 | | 0.20431 |  |
| 15 | 4264 | 20.95376 | 0.040168 | 0.102585 | 0.065844 | 0.11921 | 0.228294 | | 0.226688 |  |
| 16 | 3718 | 19.19297 | 0.000001 | 0.094974 | 0.000001 | 0.109396 | 0.218899 | | 0.226513 |  |
| 17 | 3694 | 16.59841 | 0.08458 | 0.116194 | 0.000001 | 0.113448 | 0.098785 | | 0.177625 |  |
| 18 | 3183 | 14.21144 | 0.009476 | 0.099987 | 0.008911 | 0.094594 | 0.000001 | | 0.18239 |  |
| 19 | 3267 | 11.39152 | 0.19888 | 0.133384 | 0.000001 | 0.107831 | 0.000001 | | 0.153113 |  |
| 20 | 3092 | 12.32503 | 0.146263 | 0.115584 | 0.169514 | 0.129573 | 0.240458 | | 0.199268 |  |
| 21 | 2918 | 11.42769 | 0.079527 | 0.112019 | 0.000001 | 0.095362 | 0.000001 | | 0.152639 |  |
| 22 | 3967 | 16.04925 | 0.182642 | 0.134522 | 0.156089 | 0.160669 | 0.069879 | | 0.167565 |  |
| 23 | 1893 | 8.287634 | 0.234831 | 0.125326 | 0.218615 | 0.126473 | 0.087601 | | 0.143553 |  |
| 24 | 889 | 5.378567 | 0.187412 | 0.097663 | 0.036795 | 0.073847 | 0.02865 | | 0.098663 |  |
| 25 | 1979 | 7.468873 | 0.015279 | 0.102136 | 0.000001 | 0.101571 | 0.000001 | | 0.150242 |  |
| 26 | 1769 | 6.553313 | 0.000001 | 0.085159 | 0.000001 | 0.079122 | 0.185836 | | 0.160497 |  |
| 27 | 624 | 4.365868 | 0.076935 | 0.061381 | 0.000001 | 0.072001 | 0.000001 | | 0.095577 |  |
| 28 | 1677 | 7.205943 | 0.032128 | 0.089099 | 0.107699 | 0.103377 | 0.01466 | | 0.115154 |  |
| 29 | 1144 | 6.503761 | 0.000001 | 0.057167 | 0.000001 | 0.064272 | 0.045937 | | 0.085903 |  |
| 30 | 1210 | 6.489668 | 0.000001 | 0.0682 | 0.000001 | 0.081123 | 0.000001 | | 0.10848 |  |
| Z | 3451 | 71.2484 | 0.325229 | 0.136899 | 0.000001 | 0.063757 | 0.000001 | | 0.120878 |  |

**Table S3 (cont.): Proportion of phenotypic variance (h^2^) explained by each *Sturnus vulgaris* chromosome** across the seven phenotypic traits.

| Chr | SNPs | Size (Mb) | Beak | | Wing | | Spleen | |
| --- | --- | --- | --- | --- | --- | --- | --- | --- |
|  |  |  | *V(G)/V(P)* | SE | *V(G)/V(P)* | SE | *V(G)/V(P)* | SE |
| All |  | 1029.606 | 0.000001 | 0.372156 | 0.000001 | 0.200738 | 0.999996 | 0.687702 |
| 2 | 11146 | 126.122 | 0.376227 | 0.266151 | 0.000001 | 0.155469 | 0.606218 | 0.344244 |
| 3 | 6559 | 73.37754 | 0.099251 | 0.167382 | 0.000001 | 0.135191 | 0.277022 | 0.21829 |
| 4 | 12692 | 151.9278 | 0.000001 | 0.201074 | 0.000001 | 0.150255 | 0.586597 | 0.329692 |
| 5 | 10250 | 107.3521 | 0.045721 | 0.173217 | 0.110587 | 0.18171 | 0.220664 | 0.226783 |
| 6 | 6577 | 72.52561 | 0.005565 | 0.129723 | 0.139416 | 0.16544 | 0.999999 | 0.317752 |
| 7 | 3685 | 22.39173 | 0.000001 | 0.10528 | 0.047551 | 0.113456 | 0.986031 | 0.237924 |
| 8 | 7676 | 58.5824 | 0.000001 | 0.170871 | 0.000001 | 0.134974 | 0.999998 | 0.39723 |
| 9 | 4633 | 35.15055 | 0.000001 | 0.128105 | 0.000001 | 0.105377 | 0.77921 | 0.264462 |
| 10 | 4528 | 38.36043 | 0.000001 | 0.151757 | 0.000001 | 0.10908 | 0.000001 | 0.133962 |
| 11 | 4688 | 31.01029 | 0.000001 | 0.175005 | 0.000001 | 0.128672 | 0.083782 | 0.156697 |
| 12 | 4827 | 25.75342 | 0.000001 | 0.1233 | 0.008045 | 0.105373 | 0.072053 | 0.132984 |
| 13 | 3034 | 19.02258 | 0.000001 | 0.116473 | 0.054226 | 0.100213 | 0.06276 | 0.117897 |
| 14 | 3928 | 22.3777 | 0.000001 | 0.126129 | 0.000001 | 0.11229 | 0.363798 | 0.208594 |
| 15 | 4264 | 20.95376 | 0.000001 | 0.125008 | 0.000001 | 0.113306 | 0.307447 | 0.196595 |
| 16 | 3718 | 19.19297 | 0.015257 | 0.112156 | 0.000001 | 0.122172 | 0.259599 | 0.170345 |
| 17 | 3694 | 16.59841 | 0.000001 | 0.110895 | 0.000001 | 0.098188 | 0.170543 | 0.149537 |
| 18 | 3183 | 14.21144 | 0.000001 | 0.114653 | 0.000001 | 0.086055 | 0.1574 | 0.140089 |
| 19 | 3267 | 11.39152 | 0.000001 | 0.125212 | 0.000001 | 0.111771 | 0.449739 | 0.202066 |
| 20 | 3092 | 12.32503 | 0.000001 | 0.135824 | 0.036881 | 0.104608 | 0.053795 | 0.137258 |
| 21 | 2918 | 11.42769 | 0.000001 | 0.109234 | 0.000001 | 0.105409 | 0.518085 | 0.200636 |
| 22 | 3967 | 16.04925 | 0.238601 | 0.164535 | 0.043044 | 0.113255 | 0.50594 | 0.221617 |
| 23 | 1893 | 8.287634 | 0.000001 | 0.094312 | 0.000001 | 0.079231 | 0.342609 | 0.160199 |
| 24 | 889 | 5.378567 | 0.000001 | 0.086336 | 0.000001 | 0.074629 | 0.089677 | 0.108318 |
| 25 | 1979 | 7.468873 | 0.000001 | 0.107442 | 0.042504 | 0.097254 | 0.245275 | 0.156756 |
| 26 | 1769 | 6.553313 | 0.000001 | 0.08958 | 0.149871 | 0.111092 | 0.541578 | 0.148242 |
| 27 | 624 | 4.365868 | 0.07371 | 0.079113 | 0.000001 | 0.070635 | 0.014008 | 0.082008 |
| 28 | 1677 | 7.205943 | 0.130415 | 0.107558 | 0.005869 | 0.076255 | 0.005242 | 0.099026 |
| 29 | 1144 | 6.503761 | 0.000001 | 0.063719 | 0.050149 | 0.059624 | 0.017474 | 0.07421 |
| 30 | 1210 | 6.489668 | 0.000001 | 0.092944 | 0.000001 | 0.062684 | 0.148547 | 0.102091 |
| Z | 3451 | 71.2484 | 0.000001 | 0.079125 | 0.174348 | 0.117159 | 0.000001 | 0.078433 |

**Table S3 (cont.): Proportion of phenotypic variance (h^2^) explained by each *Sturnus vulgaris* chromosome** across the seven phenotypic traits.

| Chr | SNPs | Size (Mb) | Heart | |
| --- | --- | --- | --- | --- |
|  |  |  | *V(G)/V(P)* | SE |
| All |  | 1029.606 | 0.318271 | 0.458576 |
| 2 | 11146 | 126.122 | 0.000001 | 0.169355 |
| 3 | 6559 | 73.37754 | 0.208501 | 0.188449 |
| 4 | 12692 | 151.9278 | 0.221458 | 0.254485 |
| 5 | 10250 | 107.3521 | 0.064556 | 0.191102 |
| 6 | 6577 | 72.52561 | 0.039061 | 0.145526 |
| 7 | 3685 | 22.39173 | 0.003347 | 0.107118 |
| 8 | 7676 | 58.5824 | 0.104373 | 0.174421 |
| 9 | 4633 | 35.15055 | 0.176872 | 0.170873 |
| 10 | 4528 | 38.36043 | 0.248779 | 0.189822 |
| 11 | 4688 | 31.01029 | 0.097754 | 0.158678 |
| 12 | 4827 | 25.75342 | 0.000001 | 0.130683 |
| 13 | 3034 | 19.02258 | 0.000001 | 0.111788 |
| 14 | 3928 | 22.3777 | 0.000001 | 0.11772 |
| 15 | 4264 | 20.95376 | 0.168549 | 0.150014 |
| 16 | 3718 | 19.19297 | 0.076115 | 0.139002 |
| 17 | 3694 | 16.59841 | 0.000001 | 0.119481 |
| 18 | 3183 | 14.21144 | 0.000001 | 0.096829 |
| 19 | 3267 | 11.39152 | 0.000001 | 0.120163 |
| 20 | 3092 | 12.32503 | 0.183262 | 0.148522 |
| 21 | 2918 | 11.42769 | 0.000001 | 0.111141 |
| 22 | 3967 | 16.04925 | 0.389345 | 0.214169 |
| 23 | 1893 | 8.287634 | 0.058943 | 0.10216 |
| 24 | 889 | 5.378567 | 0.000001 | 0.076951 |
| 25 | 1979 | 7.468873 | 0.024423 | 0.094994 |
| 26 | 1769 | 6.553313 | 0.083974 | 0.112972 |
| 27 | 624 | 4.365868 | 0.047678 | 0.078771 |
| 28 | 1677 | 7.205943 | 0.000001 | 0.07054 |
| 29 | 1144 | 6.503761 | 0.000001 | 0.063004 |
| 30 | 1210 | 6.489668 | 0.000001 | 0.079701 |
| Z | 3451 | 71.2484 | 0.000001 | 0.091999 |


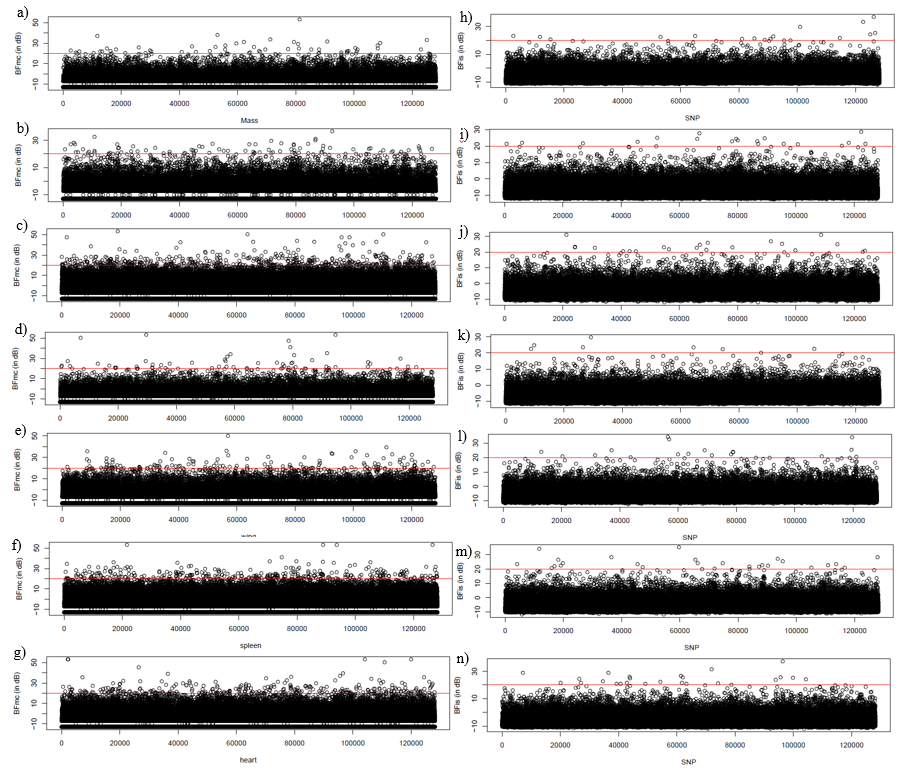


**Figure S5: BayPass generated Bayesan Factor scores** using auxmodel and anacovis for the phenotypic trait PCA axis.


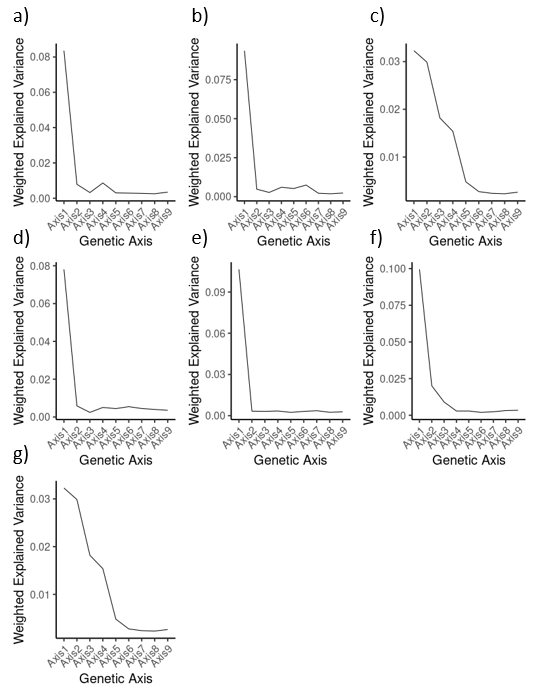


**Figure S6: Weighted overall R^2^ of the first nine PCA axis of phenotype-associated SNP groups in *Sturnus vulgaris*** for phenotype measures of a) mass, b) tarsus length, c) head antero-posterior cross section, d) beak surface area, e) wing length, f) spleen mass, and g) heart mass.


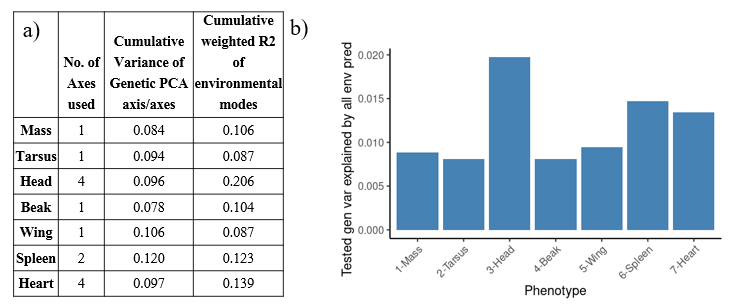


**Figure S7: Phenotype-Genetic correlations, and environmental predictors across the Australian *Sturnus vulgaris* range**. Panel a) depicts the total number of genetic PCA axis used in each analysis, as well as the cumulative genetic variance of the PCA axes and the cumulative weighted R^2^ of the environmental predictors for each of the analysis, and panel b) depicts the total proportion of variance explained by all environmental predictors for the phenotype correlated SNPs.

**Table S4: Genes flagged by BayPass analysis as phenotype-associated and also under selection in Australian *Sturnus vulgaris****.* Gene descriptions retrieved from GeneCards (Stelzer et al., 2016) on 2021/06/03.

| **Chromosome** | **Position** | **Protein Name** | **Protein Description** |
| --- | --- | --- | --- |
| **Mass** |  |  |  |
| starling20 | 11146099 | *GALK1*: galactokinase 1 | Provides instructions for making an enzyme called galactokinase 1. This enzyme enables the body to process a simple sugar called galactose, which is present in small amounts in many foods. |
| **Tarsus** |  |  |  |
| starling4 | 55648203 | *Sec61*: Protein transport protein SEC61 | Is the major component of a channel-forming translocon complex that mediates protein translocation across the endoplasmic reticulum (ER). |
| **Head** |  |  |  |
| - |  |  |  |
| **Beak** |  |  |  |
| - |  |  |  |
| **Wing** |  |  |  |
| starling22 | 1453052 | *Pigu*: Phosphatidylinositol glycan anchor biosynthesis class U protein | The protein encoded by this gene shares similarity with Saccharomyces cerevisiae Cdc91, a predicted integral membrane protein that may function in cell division control. |
| **Spleen** |  |  |  |
| starling4 | 15584986 | *Scn5a*: Sodium channel protein type 5 subunit alpha | Voltage-gated sodium channels are transmembrane glycoprotein complexes composed of a large alpha subunit with 24 transmembrane domains and one or more regulatory beta subunits. They are responsible for the generation and propagation of action potentials in neurons and muscle. This gene encodes one member of the sodium channel alpha subunit gene family. It is expressed in skeletal muscle, |
| **Heart** |  |  |  |
| starling3 | 13058973 | *LHFPL3*: L  HFPL tetraspan subfamily member 3 protein | This gene is a member of the lipoma HMGIC fusion partner (LHFP) gene family, which is a subset of the superfamily of tetraspan transmembrane protein encoding genes. Mutations in one LHFP-like gene result in deafness in humans and mice, and a second LHFP-like gene is fused to a high-mobility group gene in a translocation-associated lipoma. |
| starling25 | 291456 | *CSMD2*: CUB and sushi domain-containing protein 2 | The protein encoded by this gene is thought to be involved in the control of complement cascade of the immune system. |

**References:**

Guillerme, T., Cooper, N., Brusatte, S. L., Davis, K. E., Jackson, A. L., Gerber, S., et al. (2020). Disparities in the analysis of morphological disparity. *Biology Letters* 16, 20200199. doi:10.1098/rsbl.2020.0199.

Oksanen, J., Blanchet, F. G., Friendly, M., Kindt, R., Legendre, P., McGlinn, D., et al. (2019). *vegan: Community Ecology Package*. Available at: https://CRAN.R-project.org/package=vegan [Accessed June 8, 2020].

Pembleton, L. W., Cogan, N. O. I., and Forster, J. W. (2013). StAMPP: an R package for calculation of genetic differentiation and structure of mixed-ploidy level populations. *Mol Ecol Resour* 13, 946–952. doi:10.1111/1755-0998.12129.

Stelzer, G., Rosen, N., Plaschkes, I., Zimmerman, S., Twik, M., Fishilevich, S., et al. (2016). The GeneCards Suite: From Gene Data Mining to Disease Genome Sequence Analyses. *Curr Protoc Bioinformatics* 54, 1.30.1-1.30.33. doi:10.1002/cpbi.5.

Stuart, K. C., Cardilini, A. P. A., Cassey, P., Richardson, M. F., Sherwin, W. B., Rollins, L. A., et al. (2021). Signatures of selection in a recent invasion reveal adaptive divergence in a highly vagile invasive species. *Molecular Ecology* 30, 1419–1434. doi:10.1111/mec.15601.
